# Functional conservation of mitochondrial RNA levels despite divergent mtDNA organization

**DOI:** 10.1186/s13104-020-05177-0

**Published:** 2020-07-11

**Authors:** James P. Held, Maulik R. Patel

**Affiliations:** 1 Interdisciplinary Graduate Program, Vanderbilt University, Nashville, TN 37232 USA; 2 Department of Biological Sciences, Vanderbilt University, Nashville, TN 37232 USA; 3 Department of Cell and Developmental Biology, Vanderbilt University, Nashville, TN 37232 USA; 4 Diabetes Research and Training Center, Vanderbilt University, Nashville, TN 37232 USA

**Keywords:** Mitochondria, mtDNA, Ribosomal RNA, Droplet digital PCR, *Caenorhabditis elegans*

## Abstract

**Objective:**

Mitochondria-encoded ribosomal RNA (rRNA) genes in humans are expressed at a higher rate than protein coding genes of the mitochondria. The organization of the human mitochondrial genome (mtDNA) is amenable to differential expression of rRNAs as the rRNA encoding genes lie in tandem immediately downstream of the promoter-containing region. However, mtDNA is not organized in the same way as humans in all metazoans. In the nematode, *Caenorhabditis elegans*, the rRNA genes are on opposite sides of the mtDNA molecule and there are no obvious promoter sequences specific to the rRNA genes. Thus, we asked whether rRNA levels are higher relative to mRNAs in mitochondria of *C. elegans* as they are in humans.

**Results:**

Using droplet digital PCR, we discovered that steady-state mitochondrial rRNA transcript levels are approximately 120 times higher than the levels of mitochondrial mRNAs. These data demonstrate that despite the lack of conservation in mitochondrial genome organization, a high mitochondrial rRNA-to-mRNA ratio is a conserved feature of metazoans.

## Introduction

**Fig. 1:**
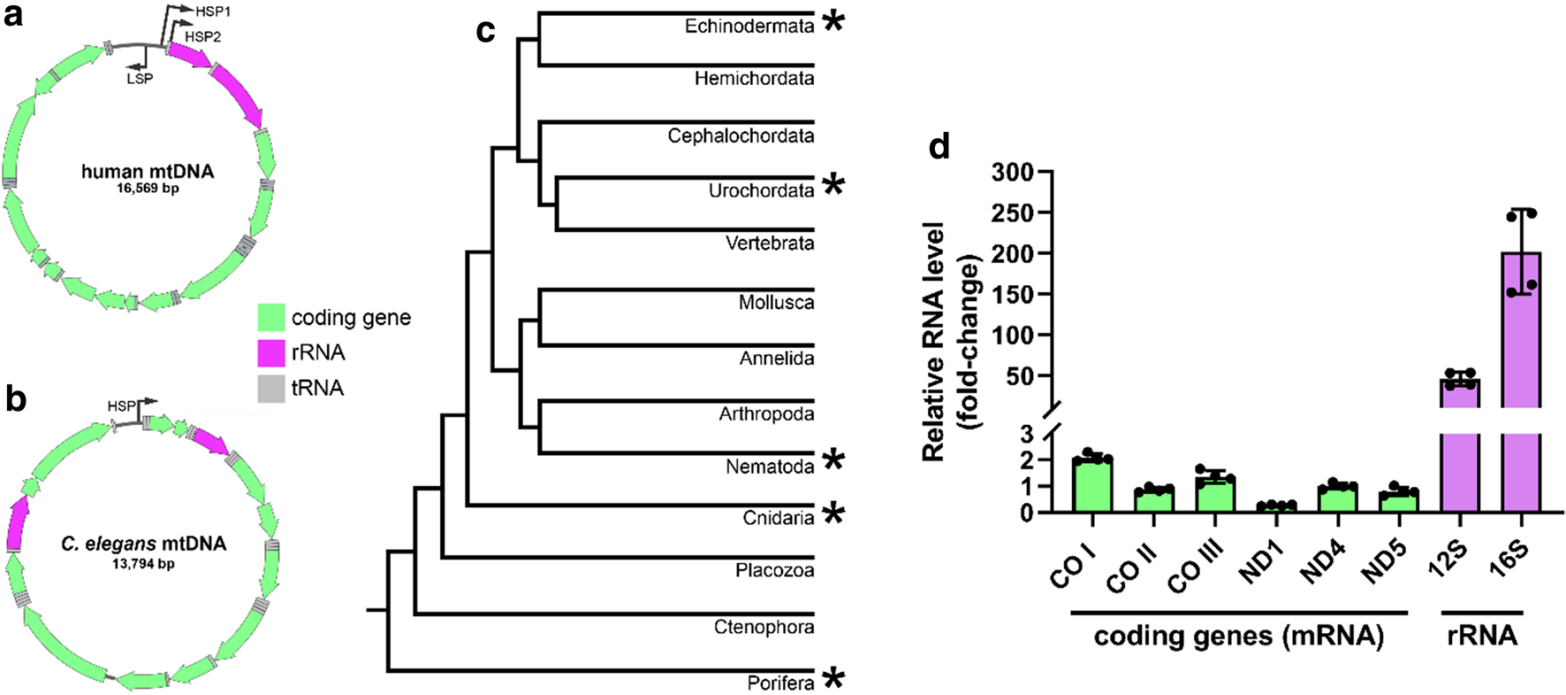
*C. elegans* has high levels of mitochondrial rRNA despite their mtDNA organization: **a** Schematic of human mitochondrial DNA (mtDNA). Human mtDNA encodes 13 essential components of the electron transport chain (depicted in green), 22 tRNAs (gray) and 2 rRNAs (magenta) required for mitochondrial translation. Human mtDNA contains three promoters: heavy strand promoter 1 (HSP1) drives expression of the rRNAs independently from the rest of the genome; heavy strand promoter 2 (HSP2) drives expression of the entire mtDNA heavy strand as a polycistronic transcript; light strand promoter (LSP) drives the transcription of the entire mtDNA light strand. **b** Schematic of *C. elegans* mtDNA. All of the genes in *C. elegans* mtDNA are presumed to be transcribed from a single heavy strand promoter (HSP) as a single polycistronic transcript. **c** Metazoan phylogeny. Asterisks indicate taxa containing species with mtDNA organization similar to *C. elegans* (rRNA encoding genes are distant from each other and mtDNA transcription occurs from a single apparent promoter). Tree branch length is arbitrary. **d** Relative RNA levels of *C. elegans* mitochondrial genes normalized to ND4 levels (n = 4 for each of the genes. COI: $\bar{x}$ = 2.07, SD = 0.15; COII: $\bar{x}$ = 0.86, SD = 0.09; COIII: $\bar{x}$ = 1.35, SD = 0.24; ND1: $\bar{x}$ = 0.27, SD = 0.03; ND4: $\bar{x}$ = 1.00, SD = 0.11; ND5: $\bar{x}$ = 0.80, SD = 0.16; 12S: $\bar{x}$ = 45.61, SD = 8.71; 16S: $\bar{x}$ = 201.7, SD = 52.24)

As some of the most ancient and essential structural components of ribosomes, rRNAs constitute the majority of total cellular RNA in both prokaryotes and eukaryotes. Mitochondria contain their own ribosomes, which are, in part, composed of two rRNAs encoded by the organellar genome, mtDNA. In humans, genes encoded by mtDNA are transcribed as polycistrons, which are subsequently processed into discrete transcripts [1]. Higher mitochondrial rRNA levels relatively to mRNA are in part accounted for by about 2.5 times higher stability of structured rRNAs [2]. Additionally, in humans, mitochondrial rRNAs are calculated to be synthesized at a rate 50 to 100 times higher than for mRNAs [2]. Higher expression of mitochondrial rRNAs relative to mRNAs appears to be made possible by the organization of mtDNA [2, 3]. In humans, the two rRNA encoding genes are positioned in tandem, and lie directly downstream of a promoter-containing region [4] (Fig. 1a). This arrangement is thought to allow the rRNA genes to be transcribed independently from their own promoter called heavy strand promoter 1 (HSP1) [4–6]. Termination of the resulting polycistron immediately downstream of the second rRNA gene ensures differential expression of the rRNAs from the rest of mtDNA transcripts encoded on the heavy strand [7, 8]. A second promoter called HSP2 is believed to regulate transcription of a polycistron that corresponds to almost the entire length of mtDNA. While there may be some controversy surrounding this “two-promoter” hypothesis [9], it is clear that the mitochondrial rRNAs are synthesized at a higher rate than the mRNA genes. However, mtDNA organization is not the same across all metazoans. A notable example is that of the model species *Caenorhabditis elegans*, in which the two rRNA encoding genes in mtDNA are separated by protein coding genes and tRNAs, making it difficult to transcribe them independently from the rest of the genome [10] (Fig. 1b). Organization of mtDNA, similar to *C. elegans*, in which the rRNAs are distant from each other and not near any rRNA-specific promoters is seen in species across metazoan taxa (phylogeny adapted from [11] (Fig. 1c). Here, we ask whether, and to what extent, *C. elegans* have a high mitochondrial rRNA-to-mRNA ratio.

## Main text

### Materials and methods

#### Worm strains

N2 (*C. elegans* wild isolate) animals were maintained at 20 °C on standard nematode growth media (NGM) plates seeded with OP50 *E. coli* bacteria.

#### RNA extraction

For each biological replicate, four 60 mm NGM plates of mixed-stage N2 worms were washed off of plates with M9 buffer into a single 15 mL conical tube. Worm suspensions were spun-down at 1300*g* for 5 min, supernatants were aspirated, and worm pellets were transferred to 2 ml RNase-free micro-centrifuge tube. Worm pellets were washed with 1 ml of M9 buffer two additional times to remove residual bacteria. Worm pellets (~ 100 μl) were snap frozen in liquid nitrogen. RNA was extracted using TRIzol™ (Thermo Scientific #15596026) as per manufacturer’s directions.

#### cDNA synthesis

cDNA was synthesized using Maxima H Minus First Strand cDNA Synthesis Kit (Thermo Scientific #K1681) following manufacturer’s directions. Briefly, ~ 500 ng of RNA from each biological replicate was combined with 1 μl of dsDNase, 1 μl of 10 × dsDNase buffer and brought-up to 10ul with nuclease-free H_2_O. This mixture was incubated at 37 °C for 2 min and then immediately returned to ice. Then, to the RNA containing tube, 1 μl random hexamer primers, 1 μl of 10 mM dNTP, 4 μl 5x RTase buffer, 1 μl of Maxima H Minus reverse transcriptase, and 3 μl of nuclease-free H_2_O were added. The cDNA synthesis reaction was run in a thermocycler: 25 °C for 10 min, 55 °C for 30 min, 85 °C for 2 min, 4 °C hold. Following cDNA synthesis, cDNA was diluted to 50 μl with nuclease-free H_2_O and stored at − 80 °C.

#### Droplet digital PCR (ddPCR)

cDNA dilution: cDNA was diluted in nuclease-free water to run in the dynamic range of ddPCR. Initially, all targets were diluted 1:200. This dilution fell within the dynamic range for the protein-genes. However, at this dilution the rRNA levels saturated the capacity of ddPCR. From this initial result it was determined that a 100x further dilution would be sufficient to accurately quantify rRNA levels. Consequently, cDNA was diluted 1:20,000 for reactions that measured rRNA targets.

ddPCR reaction: In the wells of a Eppendorf™ 96-Well twin.tec™ PCR Plate (Fisher Scientific # E951020303), 2 μl of diluted cDNA was combined with 10 μl of nuclease-free water, 12.5 μl of QX200™ ddPCR™ EvaGreen Supermix (Bio-Rad #1864034), and 0.25 μl of each corresponding forward and reverse primer (10 μM working stock). Droplets were generated on a QXDx Automated Droplet Generator. Following droplet generation cDNA was amplified: 95 °C for 5 min, 39 cycles of 95 °C for 30 s then 58 °C for 1 min, 4 °C for 5 min, 90 °C for 5 min, 10 °C hold. Following cDNA amplification, droplets were read on a QXDx Droplet Reader.

Primers: 12S rRNA: CTTGTTCCAGAATAATCGGCTAGACTTG/CTAACCAGGTACTAATCTGCTTTGTTCAAC

16S rRNA: CAGTCTTAGCGTGAGGACATTAAGGTA/CTAACCAATAACTTCATTCATACTGGAACTC

COI: CAGCAGGGTTAAGATCTATCTTAGGTGG/CGATCAGTTAACAACATAGTAATAGCCCC

COII: CTAGATCAATTAAGTTTAGGTGAACCACG/CCAAGCATGAATAACATCAGCAGATG

COIII: GCTTGAGGTAAGGATATTGCTATAGAAGG/GTGTACTGGTACTAGAGCAGCATC

ND1: GCCATCCGTGCTAGAAGACAAAG/CCTCTAACTAACTCCCTTTCACCTTCAG

ND4: ATTTCCAATTTATTTTTTACATCTTTGATTACC/CCCGCTGTGCCTAATTTTAATAG

ND5: GATCTTGGTTACCCAAAGCTATAAGAGC/GTGTCCTCAAGGCTACCACCTTCTTC

## Results and discussion

To determine the level of mitochondrial rRNAs and mRNAs in *C. elegans* we extracted RNA from mixed-stage populations of wildtype worms followed by first strand cDNA synthesis using random hexamer priming. We then used highly sensitive quantitative droplet digital PCR (ddPCR) to measure mitochondrial mRNA and rRNA steady-state levels. Upon initial quantification, the rRNA transcript counts exceeded the dynamic range of ddPCR while the mRNA levels did not. In fact, cDNA had to be diluted 100-fold further to precisely quantify rRNA levels. Using a generalized linear mixed effects model in R (package = ImerTest) we compared mitochondrial rRNA levels to mitochondrial mRNA levels after log-transformation of RNA copy number to achieve a normal distribution. We treated RNA type as a fixed effect and replicate and gene as random effects, to give a model of log (relative copy number) ~ rna type + (1|replicate) + (1|gene). Our analysis revealed that rRNA species levels are 122 times higher than mRNA species levels, after accounting for variance associated with the random effects. A Type II ANOVA on the fixed effect, using Satterthwaite’s method to approximate the degrees of freedom from the mixed model, provided an estimated F = 56.7 and p = 0.00028 (Fig. 1d).

Our finding that *C. elegans* mitochondrial rRNA levels are over 100 times higher than mitochondrial mRNAs suggests that a high mitochondrial rRNA-to-mRNA ratio is evolutionarily conserved. Furthermore, our finding suggests that multiple strategies have been employed across evolution to ensure a high mitochondrial rRNA-to-mRNA ratio. In humans, and many other metazoans, mtDNA organization provides the means for differential expression of the mitochondrial rRNA genes. However, the rRNA genes in *C. elegans* are separated by coding genes and tRNA genes and there are no apparent promoter sequences directly upstream of rRNA genes. Thus, the mechanisms to achieve high mitochondrial rRNA-to-mRNA ratio in humans are likely not available in *C. elegans,* forcing it to rely on potentially different mechanisms. One explanation is that *C. elegans* mtDNA contains cryptic promoter sequences within coding genes upstream of rRNA genes to facilitate specific expression of rRNAs. This has been proposed as a potential explanation for higher mitochondrial rRNA levels in sea urchins (*S. purpuratus*) [12]. However, this model is not consistent with the report of a single transcriptional start site in *C. elegans* mtDNA [13]. Alternatively, the post-transcriptional stability of rRNAs could be higher than that of mRNAs. Indeed, it has been shown that the half-lives of mitochondrial rRNAs in human cell lines are longer than that of mitochondrial mRNAs but not long enough to fully account for the difference in rRNA and mRNA levels [2]. Such increased stability of rRNAs may be a product of their secondary structure or a result of rRNA association with ribosomal proteins or other RNA-binding proteins. Furthermore, we cannot exclude the possibility that other post-transcriptional mechanisms are involved in facilitating high mitochondrial rRNA levels. Future investigations promise to reveal insights into mitochondrial RNA-level regulation. Nevertheless, our finding that *C. elegans* mitochondrial rRNA levels are much higher than mitochondrial mRNAs strongly suggests that a high mitochondrial rRNA-to-mRNA ratio is evolutionarily conserved and is not restrained by mtDNA organization. We propose that *C. elegans* can be used as a complimentary model system to gain insights into the mechanisms employed to maintain a high mitochondrial rRNA-to-mRNA ratio.

## Limitations

The current study reports the observation that rRNA levels are higher than mRNA levels in mitochondria. Determining the mechanism underlying this difference will have to await future studies.

## Data Availability

All data generated and analyzed during this study are included in this published article.

## Abbreviations

mtDNA: Mitochondrial DNA
rRNA: Ribosomal RNA
ddPCR: Droplet digital PCR
HSP: Heavy strand promoter
HSP1: Heavy strand promoter 1
HSP2: Heavy strand promoter 2
LSP: Light strand promoter
